# Simplification and optimization of transcatheter aortic valve implantation – fast-track course without compromising safety and efficacy

**DOI:** 10.1186/s12872-018-0976-0

**Published:** 2018-12-10

**Authors:** Manik Chopra, Ngai H. V. Luk, Ole De Backer, Lars Søndergaard

**Affiliations:** The Heart Center, Rigshospitalet, Blegdamsvej 9, 2100 Copenhagen, Denmark

**Keywords:** Aortic stenosis, Transcatheter aortic valve implantation, Simplification, Optimization, Fast-track course

## Abstract

Transcatheter aortic valve implantation (TAVI) has become an established therapeutic option for patients with symptomatic, severe aortic valve stenosis. Ageing of the Western and Asian population and expansion of indications for TAVI will lead to a substantial increase in the number of TAVI procedures performed worldwide within the next decades. In line with the maturation of TAVI over the past few years, there has also been a significant simplification and optimisation of the TAVI procedure. A minimalist TAVI procedure and fast-track TAVI course have been shown to have distinct advantages over the more traditional TAVI approach. The aim of this manuscript is to discuss strategies of TAVI simplification and optimization, with special focus on fast-track TAVI, without compromising safety and efficacy.

## Background

In 2002, Cribier et al. reported the first-in-human transcatheter aortic valve implantation (TAVI), which marked the beginning of a new era in the treatment of severe aortic valve stenosis (AS) [1]. In the early days, TAVI was performed in patients who were surgically inoperable or at high surgical risk [2, 3]. Pre-procedural assessment of the aortic valve was mainly done by echocardiography and TAVI was performed under general anaesthesia with transesophageal echocardiography (TEE) guidance. As the initial delivery systems were large, vascular complications were not uncommon. All these aspects – in combination with a limited operator experience – often led to complex and prolonged TAVI procedures and hospital stays.

During the last few years, procedural planning, TAVI systems, and clinical experience have dramatically improved. Evidence demonstrating the safety and efficacy of TAVI in patients at intermediate surgical risk has now become available, and trials are ongoing in patients at low surgical risk [4, 5]. This expansion of TAVI indications as well as ageing of the Western and Asian populations will lead to a substantial increase in the number of TAVI procedures performed worldwide within the next decades. Hence, there will be an increasing need for a simplified and fast-track TAVI approach without compromising safety and efficacy in order to treat these larger patient-flows at a reasonable economical cost.

The aim of this manuscript is to discuss strategies of TAVI simplification and optimization without compromising safety and efficacy – with special focus on fast-track TAVI – based on scientific data and our daily clinical experience in the Copenhagen TAVI center.

### Pre-procedural planning

A prerequisite for establishing and running a successful TAVI program is a thorough pre-procedural planning. Although cardiac imaging has a central role in the pre-procedural work-up, other aspects are also important in order to obtain an optimal clinical pathway.

#### Patient selection

Large randomized clinical trials conducted within the past few years have demonstrated equivalence and even net superiority of TAVI over surgical aortic valve replacement (SAVR) in high and intermediate risk patients [2–5].The prospect of bringing TAVI to lower risk and younger patient populations has led interventional cardiologists and cardiothoracic surgeons to dispute how far to push the limits; trials are ongoing to address this question.

**Fig. 1:**
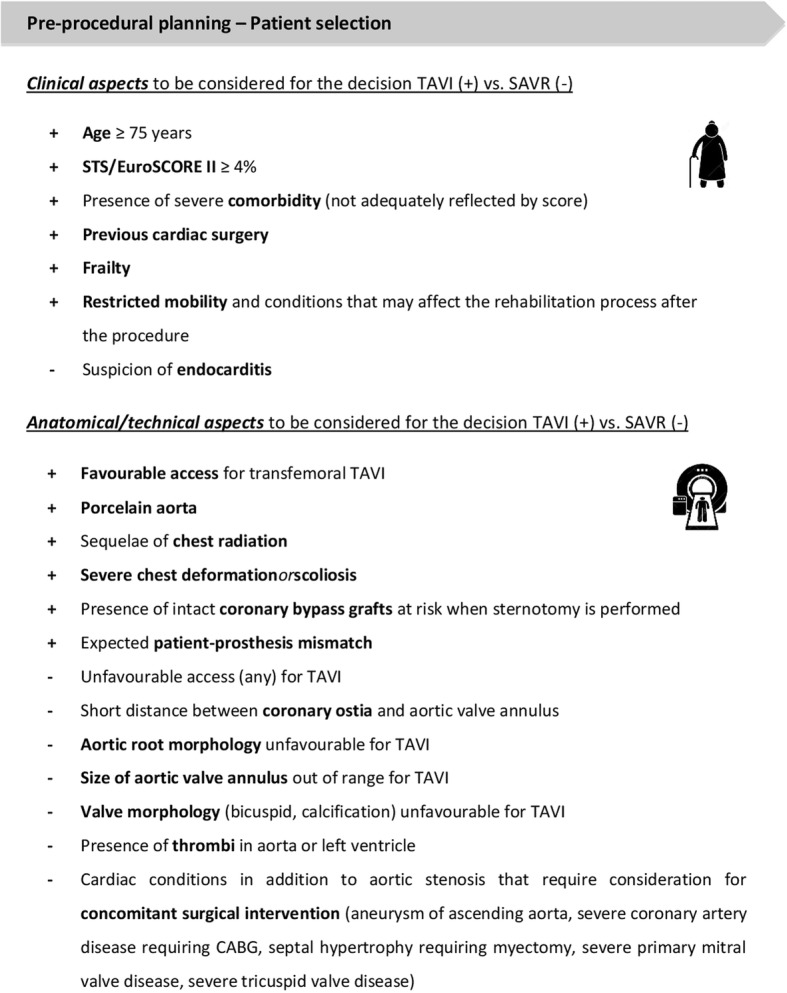
Pre-procedural planning. Clinical and anatomical/technical aspects to be considered for the decision TAVI (+) vs. SAVR (−) in the Heart Team meeting – based on the 2017 ESC/EACTS guidelines for the treatment of valvular heart disease. CABG, coronary artery bypass grafting; EACTS, European Association of Cardio-Thoracic Surgery; Euroscore II, European System for Cardiac Operative Risk Evaluation; SAVR, surgical aortic valve replacement; STS, Society of Thoracic Surgery; TAVI, transcatheter aortic valve implantation

Recently updated 2017 ESC/EACTS (European Society of Cardiology/European Association of Cardiothoracic Surgery) guidelines on the management of valvular heart disease recommend that the choice for TAVI or SAVR should not simply be based on age or a surgical risk score (STS score or EuroSCORE). A multidisciplinary Heart Team must weigh the risks and benefits of both procedures and the discussion should include multiple parameters such as age, functional status, co-morbid conditions, frailty and social support, in addition to the patient’s anatomy and the center’s outcomes for TAVI and SAVR [6]. An overview of the different clinical as well as anatomical/technical aspects to be considered are listed in Fig. 1.

#### Pre-procedural imaging

Pre-procedural imaging and assessment has dramatically improved as compared to the early days. Nowadays, the ‘gold standard’ is multi detector computed tomography (MDCT); not only to assess the aortic valve and its surrounding structures, but also to judge the possible vascular access routes.

**Fig. 2:**
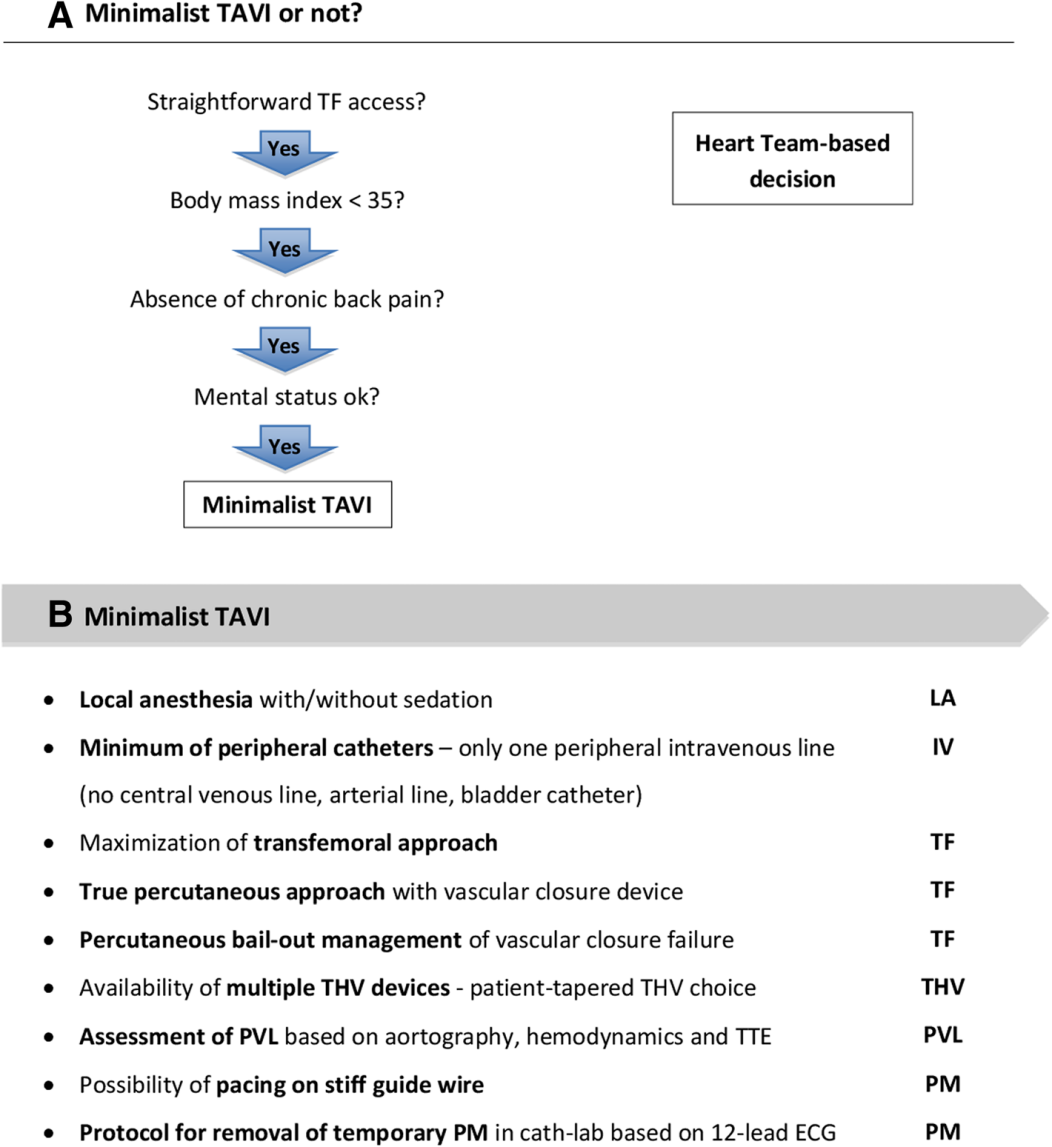
Minimalist TAVI. **a** Criteria to be considered when deciding on minimalist TAVI or not. **b** Different aspects to be considered in order to simplify the TAVI procedure. ECG, electrocardiogram; IV, intravenous; LA, local anesthesia; PM, pacemaker; PVL, paravalvular leak; TAVI, transcatheter aortic valve implantation; TF, transfemoral; THV, transcatheter heart valve; TTE, transthoracic echocardiography

In order to use MDCT as the ‘gold standard’, the acquisition and MDCT-quality has to apply to certain standards. The cardiac MDCT should be ECG-gated, contrast-enhanced and with 0.5 mm slice-thickness; the vascular access MDCT is best contrast-enhanced and with 1.0 mm slice-thickness. Based on a meticulous MDCT assessment, important considerations can be made: (1) anatomical findings that could favour SAVR or TAVI (see Fig. 1); (2) the TAVI access routes can be assessed with a preference for the transfemoral (TF) route; (3) in case different types of transcatheter heart valves (THV) are available at a center, a THV choice tailored to the patient’s anatomy can be made which may theoretically result in better outcomes. In case of alternative, non-TF access, the anatomical and technical aspects of the different access routes (subclavian, axillary, transcarotid, direct aortic, transcaval, transapical) should be discussed with the cardiothoracic surgeon.

#### Concomitant disease

When discussing a patient with symptomatic, severe AS, it is important to obtain a ‘full picture’ of the patient needing treatment. As indicated in Fig. 1, concomitant diseases or conditions may have an impact on the decision for TAVI vs. SAVR. In addition, some aspects may have an impact on the decision to plan the TAVI procedure in general anaesthesia (GA) vs. local anaesthesia (LA); e.g. severe lung disease or pulmonary hypertension, Parkinson’s disease with complex poly-pharmacy, conditions that may affect the rehabilitation process, etc.

Assessment of the coronary arteries before TAVI is also a standard practice. The ESC/EACTS guidelines recommend percutaneous coronary intervention (PCI) in patients undergoing TAVI with greater than 70% stenosis at proximal coronary segments [7].However, there is currently no conclusive evidence as to whether PCI should be performed or not, and whether a staged or hybrid intervention should be preferred. The decision should be made on an individual basis depending on the leading clinical problem, the complexity of the underlying coronary artery disease, and the presence of co-morbidities such as renal dysfunction [8].

#### Patient information

Finally, it is important that the decision taken by the Heart Team with regards to the type of therapy, anaesthesia, and route of access in case of TAVI is communicated to the patient and the relatives. Patients should be counselled about the risks/benefits of the procedure and the different steps to come. A thorough pre-procedural work-up and good patient information helps to prepare the patient for early mobilisation and early discharge (if possible).

Centres with a high volume TAVI program frequently have Transcatheter Valve Therapies (TVT) or TAVI coordinators. This function plays an important role in: (1) streamlining and prioritizing referrals for TAVI; (2) clinical assessment of the patient by means of mini-mental state examination, 6-min walk test, frailty test, etc.; (3) making appointments for MDCT and transthoracic echocardiography (TTE); and (4) coordinating care for the out- and in-patient settings. This may reduce the preparatory time required by physicians and free them for other more physician-specific tasks [9].

### Minimalist TAVI

In line with the maturation of TAVI over the past decade, there has also been a significant simplification of the TAVI procedure. A simplified or minimalist TAVI approach (Fig. 2a and b) has been shown to be as safe and effective as the more traditional approach and is nowadays routine in many centers [10–14]. Minimalist TAVI not only leads to a reduced procedural time but also a shorter intensive care unit (ICU) and hospital stay, lower resource use and lower hospital costs [10, 12, 14, 15].

#### Transfemoral approach

Maximization of TF access is one of the most important aspects when trying to maximally exploit the benefits of a minimalist TAVI approach (Fig. 2b). Nowadays, most of the TAVI procedures– even those with challenging peripheral access – can be performed by TF approach using low profile sheaths and devices [16]. In a meta-analysis by Zhao et al., TF-TAVI was reported to result in a higher 30-day and 1-year survival as compared to transapical TAVI [17]. In a meta-analysis by Siontis et al., a lower 2-year mortality rate was reported for TF-TAVI as compared to SAVR, whereas this was not true for alternative access TAVI [18]. However, interpretation of these results is difficult as transapical and alternative access TAVI groups also typically contain a larger number of patients at higher surgical risk with multiple co-morbidities.

In order to fully exploit all benefits of the TF approach, a true percutaneous approach with the use of a vascular (pre)-closure device should be adopted [19]. Routine use of ultrasound-guided vascular puncture and a safety wire help to keep major vascular complications to a minimum. In case of incomplete vascular closure or vascular closure device failure, percutaneous intervention using balloon tamponade and/or a covered stent should be part of the operator’s team therapeutic arsenal [20].

#### Local anaesthesia

As the majority of TAVI nowadays are performed by TF approach, this has also led to an increased adoption of LA or conscious sedation. GA in many centers is used only in case of alternative access. However, even though most operators are convinced that TF-TAVI is optimal when possible, some still favor GA over LA [21].

Depending on local routines, politics and/or legislation, TAVI procedures are performed with or without anaesthesia support in the cathlab. Also specific patient characteristics (e.g. extreme obesity, mental disorder, chronic backpain) may still favor the use of GA for certain TAVI procedures at our center (Fig. 2a). One of the largest advantages of performing TAVI without GA is that patients tend to be hemodynamically more stable with less need for inotropic agents. TAVI with LA only is also the better choice for patients with significant lung disease or pulmonary hypertension. Moreover, performing TAVI under LA has a distinct advantage as monitoring of the neurological status is possible thoughout the entire procedure; it also allows pain assessment which is important during certain phases of the procedure as pain can signal risk for vascular complication [22]. Several studies report that TAVI under LA is at least as safe as TAVI under GA [23–25]. Furthermore, there is a reduced procedural time, no longer need for ICU stay, and a reduced length of hospital stay; all these aspects make TAVI under LA a cost-effective alternative to TAVI under GA [23–25].

#### Paravalvular leak (PVL) assessment

As TAVI is increasingly performed in LA or conscious sedation only, use of peri-procedural TEE to assess PVL is no longer routine in many centers. The use of TEE demands GA and data supports the fact that clinical outcomes are similar for TAVI under LA without TEE vs. TAVI under GA with TEE, with LA offering other advantages as explained above [13, 14, 23–27]. In one study, TAVI with TTE only in a high-risk patient group was associated with an increased incidence of intra-procedural PVL, although this was not associated with higher rates of PVL at follow-up [28]. However, it is important not to rely on one measurement only – in order to assess the grade of PVL immediately after THV implantation, most operators take a combined evaluation of aortography, hemodynamic parameters and TTE.

#### Minimum of catheters and lines

A policy of ‘no catheters except for one peripheral venous line’ adds to the simplification of TAVI. A recent observational TAVI study suggests that avoidance of bladder catheterization minimizes in-hospital complications, with significantly lower rates of urinary infection, haematuria and need for continuous bladder irrigation [29]. Also a central venous line has its own complications and is best avoided, if possible [30].

#### Newer THV devices

Availability and use of newer THV devices may help when implementing minimalist TAVI, as these newer devices have a lower insertion profile, are often repositionable, have a more stable and predictable valve deployment, and have a lower incidence of significant PVL. It is a debated topic from which annual TAVI volume a second THV system should be introduced in a center. A difficult balance should be kept between increased experience with a specific THV vs. the opportunity to choose a specific THV type tailored to the patient’s specific anatomy. Clearly, it is an advantage if TAVI cases can be centralized in a larger volume TAVI center as this will not only result in a higher quality TAVI program, but this will also give the possibility to have different THV devices in use.

#### Pacemaker

Routine use of a balloon-tipped temporary pacemaker (PM) may reduce the risk of right ventricular perforation. Another possibility is to pace the left ventricle (LV) by using the stiff guidewire in the LV, which may further add to TAVI simplification [31]. In our center, the temporary PM is removed in the cathlab in the majority of cases after assessment of a post-TAVI ECG and with the help of a pre-specified protocol [32]. One other study also showed that patients with normal PR and QRS conduction post-TAVI did not develop delayed high-degree conduction disorders [33]. These patients are the best candidates for removal of their temporary PM in cathlab. In case of high-degree conduction disorders, switching the temporary PM from a transfemoral to a transjugular insertion with a screw-in lead helps to mobilize these patients early after the TAVI procedure.

#### TAVI team

When having the ambition to be a center of excellence, it is must to build a dedicated TAVI team involving experienced TAVI operators, cathlab nurses who are also trained in valve loading, and a TAVI coordinator. As for most interventions, a large experience is often the best guarantee for a high success rate. Also for the implementation of minimalist TAVI, it is an advantage to be able to rely on a team with large expertise in TAVI and basic peripheral vascular intervention skills. A dedicated hybrid lab is the preferred work place.

### Post-procedural care

#### Early mobilization

**Fig. 3:**
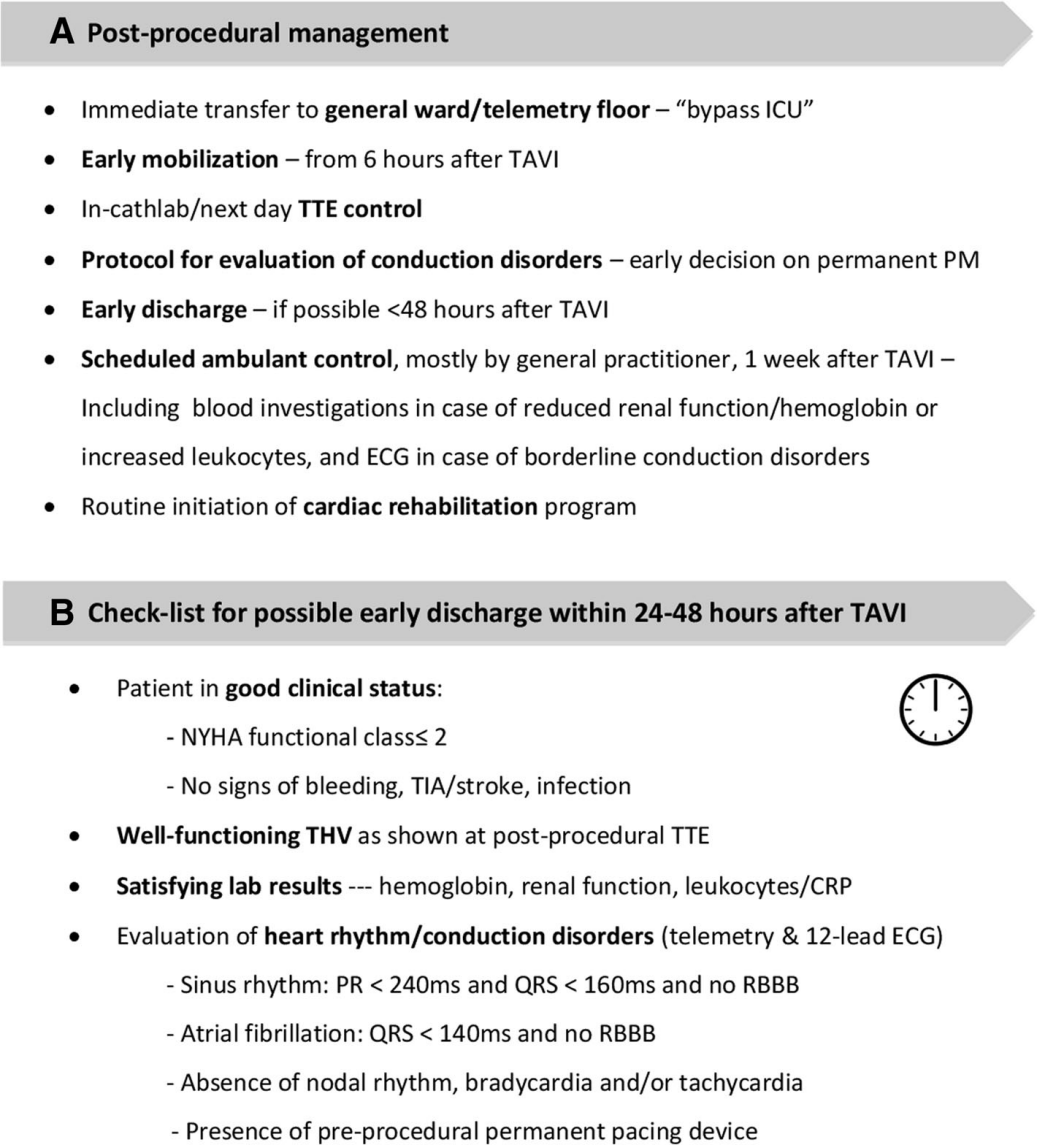
Post-procedural management. **a** Different aspects to be considered in order to optimize post-TAVI care and obtain a ‘fast-track’ TAVI course. **b** Check-list for possible early discharge within 24 to 48 h after TAVI – as applied at the Copenhagen TAVI center. CRP, C reactive protein; ECG, electrocardiogram; ICU, intensive care unit; NYHA, New York Heart Association; PM, pacemaker; RBBB, right bundle branch block; TAVI, transcatheter aortic valve implantation; THV, transcatheter heart valve; TIA, Transient ischemic attack; TTE, transthoracic echocardiography

As the majority of TAVI cases nowadays can be performed by a true percutaneous TF approach without the need for GA, most of these patients can immediately after completion of the procedure be transferred from the catheterization laboratory to the telemetry floor (Fig. 3). This not only eliminates a large ‘burden’ on the ICU, but also results in a significant cost reduction. Only those patients that underwent alternative access TAVI or those with peri-procedural complications are still monitored at ICU during the post-procedural period.

Early mobilisation from 6 h after TAVI and discharge within 24 to 48 h should be aimed for as not only does it result in cost savings, but can also reduce the risk of post-operative delirium and result in a faster improvement of the patient’s quality of life [34, 35].

#### Decision on permanent pacemaker

An internal protocol for the evaluation of conduction disorders and, hence, decision on the need for a permanent PM within 24 h after TAVI helps to reduce the hospital length of stay (LoS). In case a permanent PM is needed, implantation within 72 h after TAVI should be aimed for as this helps avoiding unnecessary prolongation of the hospitalization.

#### Length of hospitalization

**Fig. 4:**
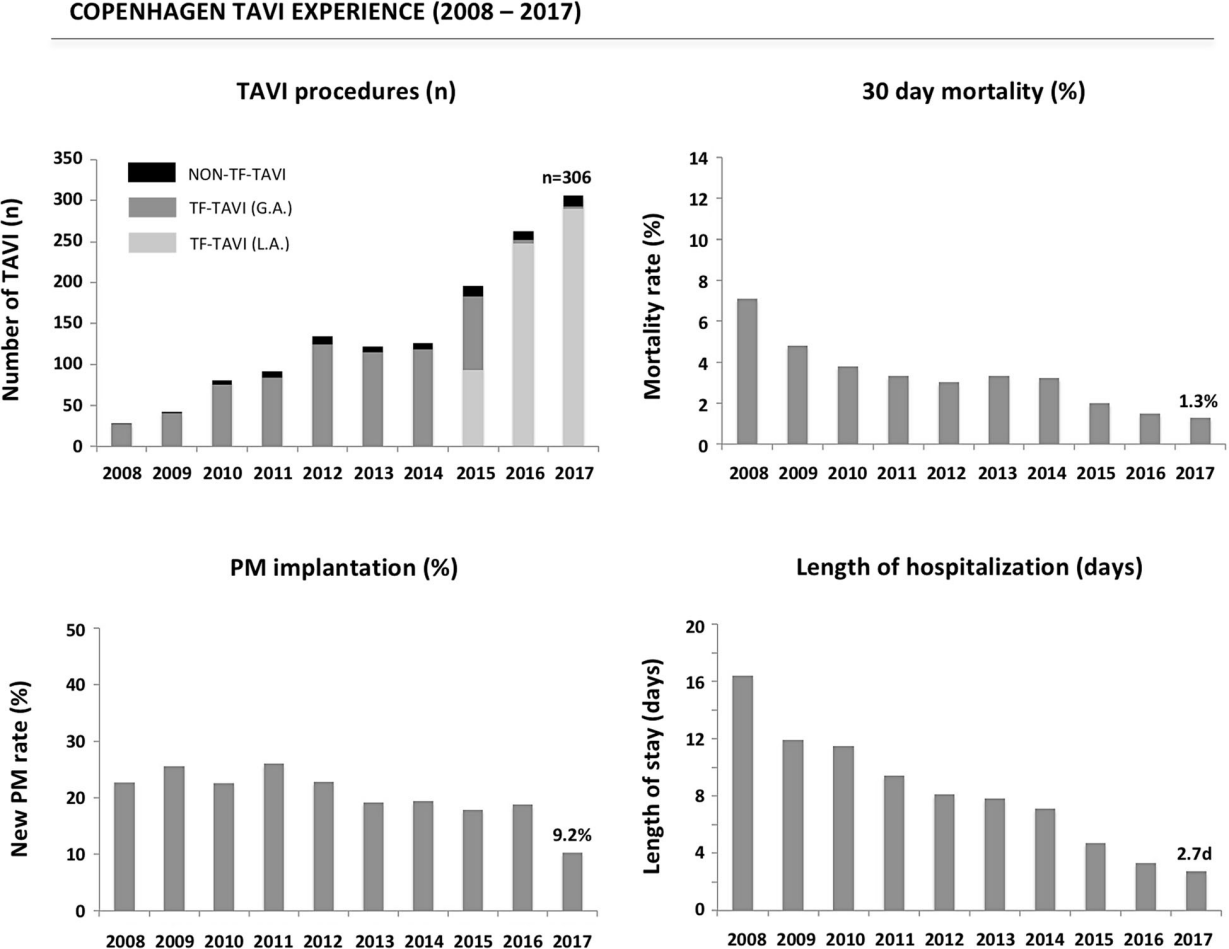
Copenhagen TAVI experience. Results from the East Denmark Heart Registry reporting on the TAVI approach and short-term clinical outcomes in the period 2008 to 2017. GA, general anaesthesia; LA, local anaesthesia; PM, pacemaker; TAVI, transcatheter aortic valve implantation; TF, transfemoral

In 2016, the Vancouver group reported on their TAVI clinical pathway, thereby implementing a minimalist TAVI approach, standardized care, and discharge criteria to reduce LoS. Between May 2012 and October 2014, 397 TF-TAVI were completed – of these, 150 (38.2%) were discharged within 48 h, whereas only 39 patients (9.9%) were hospitalized for more than 5 days [36]. In our experience in Copenhagen, 543 patients underwent TF-TAVI in the 2016–2017 period – of these 314 (57.8%) were discharged within 48 h, whereas 43 patients (7.9%) were hospitalized for more than 5 days (Fig. 4). Clearly, LoS after TAVI is not only depending on the optimization and implementation of clinical pathways, but also on the risk profile of the TAVI population and rules for reimbursement in specific countries. Importantly, a short post-procedural LoS was not associated with an increased risk of readmission within 30 days or 1 year. On the contrary, the risk of 1-year readmission increased with longer post-TAVI LoS [37]. Few other studies also suggest no difference in 30-day rehospitalisation and clinical outcomes in case of early discharge after TF-TAVI [38, 39].

#### Anti-thrombotic regimen

Currently, there is still a lot of debate and uncertainty about the optimal post-procedural anti-thrombotic treatment following TAVI. Numerous on-going trials are comparing different anti-platelet and anti-thrombotic regimens in patients undergoing TAVI. Only small studies in TAVI patients are available, which are mostly observational and likely underpowered.

The current AHA/ACC (American Heart Association/American College of Cardiology) guidelines and ESC/EACTS guidelines recommend dual antiplatelet therapy (DAPT) for 3 to 6 months followed by aspirin lifelong. However, it is generally expected that these recommendations will be further ‘simplified’ in the near future. Preliminary data seem to indicate that single antiplatelet therapy may be as safe and as effective as DAPT for patients undergoing TAVI. ESC/EACTS guidelines suggest that single antiplatelet therapy may be considered instead of DAPT in patients with high bleeding risk [6, 40, 41].

Oral anticoagulation is treatment of choice in patients who have other indications for anticoagulation like Atrial fibrillation (AF), venous thromboembolism, hypercoagulable state or, with a lesser degree of evidence, severely impaired LV dysfunction (ejection fraction < 35%) [6]. Anticoagulation with a Vitamin K antagonists (VKA) to achieve an INR of 2.5 may be reasonable for at least 3 months after TAVR in patients at low risk of bleeding [40]. In patients with AF, VKA will remain the treatment of choice. However, additional antiplatelet therapy seems only reasonable in patients with recent acute coronary syndrome, extensive or recent coronary stenting or large aortic arch atheroma. Initiation of VKA is indicated in clinical valve leaflet thrombosis, whereas the role of VKA in the case of subclinical leaflet thrombosis is currently uncertain [42].

#### Follow-up

As more than 75% of TF-TAVI patients are discharged within 72 h in Copenhagen, we routinely foresee a follow-up consultation with the general practitioner approximately one week after TAVI for all patients. In this way, patients are guaranteed a clinic visit, checking for fever (in case of infection), dizziness (in case of conduction disorders), or taking blood samples in case of reduced renal function or borderline elevated infection parameters at discharge. Routine ‘home-care’ in frail elderly patients, follow-up at a local heart failure clinic in patients with reduced LV function, and referral of all patients to a cardiac rehabilitation program adds to comprehensive patient management. In this context, an Italian study previously reported that involving TAVI patients in a cardiac rehabilitation program significantly improves their functional status, quality of life, and autonomy [43].

Concerning echocardiographic follow-up post-TAVI, the most recent European guidelines prescribe a TTE before discharge followed by echocardiographic assessment of the valve function at 1 to 3 months after TAVI, at 1 year after TAVI and annually thereafter (with additional follow-up assessment and/or integration of other imaging modalities as necessary and/or determined by the attending physician) in order to diagnose possible valve failure or valve dysfunction in time [44].

## Conclusion

Expansion of indications for TAVI and ageing of the Western/Asian population will lead to a substantial increase in the number of TAVI procedures performed worldwide within the next decades. A minimalist TAVI procedure and fast-track TAVI course have been shown to have distinct advantages over the more traditional approach without compromising safety and clinical efficacy. Moreover, this TAVI optimization creates the opportunity to reduce the burden on hospital resources and add to cost-effectiveness when confronted with a growing population of patients with severe AS.

## Abbreviations

ACC: American College of Cardiology
AF: Atrial fibrillation
AHA: American Heart Association
AS: Aortic valve stenosis
CABG: Coronary artery bypass grafting
CRP: C reactive protein
DAPT: Dual antiplatelet therapy
EACTS: European Association of Cardio-Thoracic Surgery
ECG: 12- lead Electrocardiogram
ESC: European Society of Cardiology
EuroSCORE: European System for Cardiac Operative Risk Evaluation
GA: General anaesthesia
ICU: Intensive care unit
IV: Intravenous
LA: Local anaesthesia
LoS: Hospital length of stay
LV: Left ventricle
MDCT: Multi detector computed tomography
NYHA: New York Heart Association
PCI: Percutaneous coronary intervention
PM: Pacemaker
PVL: Paravalvular leak
RBBB: Right bundle branch block
SAVR: Surgical aortic valve replacement
STS: Society of Thoracic Surgery
TAVI: Transcatheter aortic valve implantation
TEE: Transesophageal echocardiography
TF: Transfemoral
THV: Transcatheter heart valves
TIA: Transient ischemic attack
TTE: Transthoracic echocardiography
TVT: Transcatheter valve therapies
VKA: Vitamin K antagonist

## References

[1] Cribier A, Eltchaninoff H, Bash A, Borenstein N, Tron C, Bauer F (2002). Percutaneous transcatheter implantation of an aortic valve prosthesis for calcific aortic stenosis: first human case description. Circulation.

[2] Leon MB, Smith CR, Mack M, Miller DC, Moses JW, Svensson LG (2010). Transcatheter aortic-valve implantation for aortic stenosis in patients who cannot undergo surgery. N Engl J Med.

[3] Smith CR, Leon MB, Mack MJ, Miller DC, Moses JW, Svensson LG (2011). Transcatheter versus surgical aortic-valve replacement in high-risk patients. N Engl J Med.

[4] Leon MB, Smith CR, Mack MJ, Makkar RR, Svensson LG, Kodali SK (2016). Transcatheter or Surgical Aortic-Valve Replacement in Intermediate-Risk Patients. N Engl J Med.

[5] Reardon MJ, Van Mieghem NM, Popma JJ, Kleiman NS, Søndergaard L, Mumtaz M (2017). Surgical or Transcatheter aortic-valve replacement in intermediate-risk patients. N Engl J Med.

[6] Baumgartner H, Falk V, Bax JJ, De Bonis M, Hamm C, Holm PJ (2017). 2017 ESC/EACTS Guidelines for the management of valvular heart disease. Eur Heart J.

[7] Windecker S, Kolh P, Alfonso F, Collet JP, Cremer J, Falk V (2014). 2014 ESC/EACTS guidelines on myocardial revascularization: the task force on myocardial revascularization of the European Society of Cardiology (ESC) and the European Association for Cardio-Thoracic Surgery (EACTS)developed with the special contribution of the European Association of Percutaneous Cardiovascular Interventions (EAPCI). Eur Heart J.

[8] Goel SS, Ige M, Tuzcu EM, Ellis SG, Stewart WJ, Svensson LG (2013). Severe aortic stenosis and coronary artery disease: implications for management in the transcatheter aortic valve replacement era: a comprehensive review. J Am Coll Cardiol.

[9] Coylewright M, Mack MJ, Holmes DR, O'Gara PT (2015). A call for an evidence-based approach to the heart team for patients with severe aortic stenosis. J Am Coll Cardiol.

[10] Babaliaros V, Devireddy C, Lerakis S, Leonardi R, Iturra SA, Mavromatis K (2014). Comparison of Transfemoral transcatheter aortic valve replacement performed in the catheterisation laboratory (minimalistic approach) versus hybrid operating room (Standard approach): outcomes and cost analysis. JACC Cardiovasc Interv.

[11] Frangieh AH, Ott I, Michel J, Shivaraju A, Joner M, Mayr NP (2017). Standardized minimalistic Transfemoral Transcatheter aortic valve replacement (TAVR) using the SAPIEN 3 device: stepwise description, feasibility, and safety from a large consecutive single-center single-operator cohort. Structural Heart.

[12] Gurevich S, Oestreich B, Kelly RF, Mbai M, Bertog S, Ringsred K (2018). Outcomes of transcatheter aortic valve replacement using a minimalist approach. Cardiovasc Revasc Med.

[13] Barbanti M, Tamburino C (2015). Optimisation of TAVI: is it mature enough to be defined as a PCI-like procedure?. EuroIntervention.

[14] Jensen HA, Condado JF, Devireddy C, Binongo J, Leshnower BG, Babaliaros V (2015). Minimalist transcatheter aortic valve replacement: the new standard for surgeons and cardiologists using transfemoral access?. J Thorac Cardiovasc Surg.

[15] Ribera A, Slof J, Andrea R, Falces C, Gutiérrez E, Del Valle-Fernández R (2015). Transfemoral transcatheter aortic valve replacement compared with surgical replacement in patients with severe aortic stenosis and comparable risk: cost-utility and its determinants. Int J Cardiol.

[16] Barbanti M, Binder RK, Freeman M, Wood DA, Leipsic J, Cheung A (2013). Impact of low-profile sheaths on vascular complications during transfemoral transcatheter aortic valve replacement. EuroIntervention.

[17] Zhao A, Minhui H, Li X, Zhiyun X (2015). A meta-analysis of transfemoral versus transapical transcatheter aortic valve implantation on 30-day and 1-year outcomes. Heart Surg Forum.

[18] Siontis GC, Praz F, Pilgrim T, Mavridis D, Verma S, Salanti G (2016). Transcatheter aortic valve implantation vs. surgical aortic valve replacement for treatment of severe aortic stenosis: a meta-analysis of randomized trials. Eur Heart J.

[19] Israel M, Barbash IM, Barbanti M, Webb J, Molina-Martin De Nicolas J, Abramowitz Y (2015). Comparison of vascular closure devices for access site closure after transfemoral aortic valve implantation. Eur Heart J.

[20] Stortecky S, Wenaweser P, Diehm N, Pilgrim T, Huber C, Rosskopf AB (2012). Percutaneous management of vascular complications in patients undergoing transcatheter aortic valve implantation. JACC Cardiovasc Interv.

[21] Marcantuono R, Gutsche J, Burke-Julien M, Anwaruddin S, Augoustides JG, Jones D (2015). Rationale, development, implementation, and initial results of a fast track protocol for transfemoral transcatheter aortic valve replacement (TAVR). Catheter Cardiovasc Interv.

[22] Dvir D, Jhaveri R, Pichard AD (2012). The minimalist approach for transcatheter aortic valve replacement in high-risk patients. JACC Cardiovasc Interv.

[23] Jabbar A, Khaurana A, Mohammed A, Das R, Zaman A, Edwards R (2016). Local versus general Anaesthesia in Transcatheter aortic valve replacement. Am J Cardiol.

[24] Villablanca PA, Mohananey D, Nikolic K, Bangalore S, Slovut DP, Mathew V (2018). Comparision of local versus general anaesthesia in patients undergoing transcatheter aortic valve replacement: A meta-analysis. Catheter Cardiovasc Interv.

[25] Fröhlich GM, Lansky AJ, Webb J, Roffi M, Toggweiler S, Reinthaler M (2014). Local versus general anaesthesia for transcatheter aortic valve implantation (TAVR) – Systematic review and meta-analysis. BMC Med.

[26] Krishnaswamy A, Latib A, Malik A, Bertoldi L, Poddar KL, Chieffo A (2016). Resource utilization for transfemoral transcatheter aortic valve replacement: an international comparision. Catheter Cardiovasc Interv.

[27] Cilingiroglu M, Marmagkiolis K (2016). A glimpse into future of TAVR. Catheter Cardiovasc Interv.

[28] Hayek SS, Corrigan FE, Condado JF, Lin S, Howell S, MacNamara JP (2017). Paravalvular Regurgitation after Transcatheter Aortic Valve Replacement: Comparing Transthoracic versus Transesophageal Echocardiographic Guidance. J Am Soc Echocardiogr.

[29] Lauck SB, Kwon JY, Wood DA, Baumbusch J, Norekvål TM, Htun N (2018). Avoidance of urinary catheterization to minimize in-hospital complications after transcatheter aortic valve implantation: an observational study. Eur J Cardiovasc Nurs.

[30] Kornbau C, Lee KC, Hughes GD, Firstenberg MS (2015). Central line complications. Int J Crit Illn Inj Sci.

[31] Faurie B, Abdellaoui M, Wautot F, Staat P, Champagnac D, Wintzer-Wehekind J (2016). Rapid pacing using the left ventricular guidewire: Reviving an old technique to simplify BAV and TAVI procedures. Catheter Cardiovasc Interv.

[32] Jørgensen TH, De Backer O, Gerds TA, Bieliauskas G, Svendsen JH, Søndergaard L (2018). Immediate post-procedural 12-Lead electrocardiography as predictor of late conduction defects after Transcatheter aortic valve replacement. JACC Cardiovasc Interv..

[33] Toggweiler S, Stortecky S, Holy E, Zuk K, Cuculi F, Nietlispach F (2016). The electrocardiogram after Transcatheter aortic valve replacement determines the risk for post-procedural high-degree AV block and the need for telemetry monitoring. JACC Cardiovasc Interv.

[34] Wood DA (2016). Could a Simplified Transcatheter Aortic Valve Replacement Procedure Eliminate Post –Operative Delirium?. JACC Cardiovasc Interv.

[35] Abawi M, Nijhoff F, Agostoni P, Emmelot-Vonk MH, de Vries R, Doevendans PA (2016). Incidence, predictive factors, and effect of delirium after transcatheter aortic valve replacement. JACC Cardiovasc Interv.

[36] Lauck SB, Wood DA, Baumbusch J, Kwon JY, Stub D, Achtem L (2016). Vancouver transcatheter aortic valve replacement clinical pathway: minimalist approach, standardized care, and discharge criteria to reduce length of stay. Circ Cardiovasc Qual Outcomes.

[37] Sud M, Qui F, Austin PC, Ko DT, Wood D, Czarnecki A, et al. Short Length of Stay After Elective Transfemoral Transcatheter Aortic Valve Replacement is Not Associated With Increased Early or Late Readmission Risk. J Am Heart Assoc. 2017;6(4):e005460. 10.1161/JAHA.116.005460 PMC5533033 28438738

[38] Durand E, Eltchaninoff H, Canville A, Bouhzam N, Godin M, Tron C (2015). Feasibility and safety of early discharge after transfemoral transcatheter aortic valve implantation with the Edwards SAPIEN-XT prosthesis. Am J Cardiol.

[39] Barbanti M, Capranzano P, Ohno Y, Attizzani GF, Gulino S, Imme S (2015). Early discharge after transfemoral transcatheter aortic valve implantation. Heart.

[40] Nishimura RA, Otto CM, Bonow RO, Carabello BA, Erwin JP, Fleisher LA (2017). 2017 AHA/ACC focused update of the Management of Patients with Valvular Heart Disease: a report of the American College of Cardiology/American Heart Association task force on clinical practice guidelines. J Am Coll Cardiol.

[41] Ahmad Y, Demir O, Rajkumar C, Howard JP, Shun-Shin M, Cook C (2018). Optimal antiplatelet strategy after transcatheter aortic valve implantation: a meta-analysis. Open Heart.

[42] Søndergaard L, Sigitas C, Chopra M, Bieliauskas G, De Backer O (2017). Leaflet Thrombosis after TAVI. Eur Heart J.

[43] Zanettini R, Gatto G, Mori I, Pozzoni MB, Pelenghi S, Martinelli L (2014). Cardiac rehabilitation and mid-term follow-up after transcatheter aortic valve implantation. J Geriatr Cardiol.

[44] Capodanno D, Petronio AS, Prendergast B, Eltchaninoff H, Vahanian A, Modine T (2017). Standardized definations of structural deterioration and valve failure in assessing long-term durability of transcatheter and surgical aortic bioprostheic valves: a consensus statement from the European Association of Percutaneus Cardiovascular Interventions (EAPCI) endorsed by the European Society of Cardiology (ESC) and the European Association for Cardio-Thoracic Surgery (EACTS). Eur Heart J.

